# Pregnancy Outcomes in Patients With Adult-Onset Still's Disease: A Cohort Study From China

**DOI:** 10.3389/fmed.2020.566738

**Published:** 2020-12-08

**Authors:** Zhihong Wang, Huihui Chi, Tienan Feng, Qinwen Du, Ting Zeng, Jialin Teng, Honglei Liu, Xiaobing Cheng, Junna Ye, Hui Shi, Yue Sun, Qiongyi Hu, Jinchao Jia, Tingting Liu, Liyan Wan, Xinyao Wu, Zhuochao Zhou, Chengde Yang, Yutong Su

**Affiliations:** ^1^Department of Rheumatology and Immunology, Ruijin Hospital, Shanghai Jiao Tong University School of Medicine, Shanghai, China; ^2^Hongqiao International Institute of Medicine, Shanghai Tongren Hospital/Clinical Research Institute, Shanghai Jiao Tong University School of Medicine, Shanghai, China; ^3^Department of Obstetrics and Gynecology, Ruijin Hospital, Shanghai Jiao Tong University School of Medicine, Shanghai, China; ^4^Department of Rheumatology, Xinhua Hospital Chongming Branch Affiliated to Shanghai Jiao Tong University School of Medicine, Shanghai, China

**Keywords:** pregnancy, outcome, generalized linear mixed effect model, adverse impact, adult-onset still's disease

## Abstract

**Objective:** Adult-onset Still's disease (AOSD) is an autoinflammatory disease with a higher prevalence rate in young females. The purpose of this study is to investigate whether AOSD has an adverse impact on pregnancy outcomes, or conversely exacerbated by pregnancy.

**Methods:** The outcomes of 191 pregnancies were evaluated in 86 female patients with AOSD. The generalized linear mixed model and propensity score matching method were conducted to evaluate the influence of AOSD on pregnancy outcomes. A dependent sample sign test was applied to assess the impact of pregnancy on the relapse of AOSD.

**Results:** The results showed that the post-AOSD group had a lower proportion of normal delivery (25.0 vs. 52.4%, *p* = 0.036) and a higher proportion of spontaneous abortion (STA) (18.8 vs. 0.6%, *p* = 0.002) compared with the pre-AOSD group. Moreover, pregnancy after being diagnosed with AOSD was a significant high risk factor of STA (adjusted OR = 4.577, 95% CI: 4.166–845.119; *p* = 0.003). Disease flare upon conception was observed in one of 16 post-AOSD pregnancies (*p* = 1.000). There were 11 patients with new-onset AOSD during gestation or postpartum, among which five (45.4%) evolved into the polycyclic course.

**Conclusions:** AOSD patients might suffer from a higher risk of STA, however, pregnancy might not be related with the exacerbation of diagnosed AOSD. New-onset AOSD during gestation or postpartum tend to evolve into the polycyclic course.

## Introduction

Adult-onset Still's disease (AOSD) is a systemic disorder of unknown etiology characterized by spiking fever, arthralgia or arthritis, and evanescent rash (1, 2). AOSD is a rare autoinflammatory disease with an estimated prevalence of one to 34 cases per million people (3), and predominantly affects female at a young age (4, 5). The etiology and pathogenesis of AOSD is unknown. Our previous studies showed that CMV infections, increased neutrophil extracellular traps, and multiple other factors were involved in the development of AOSD (6–10).

In clinical practice, one of the major problems that make young female patients worried is the reproductive health condition after being diagnosed with AOSD. Currently, the relationship between pregnancy and AOSD, including disease onset and relapse (pregnancy outcomes in patients with AOSD), still remains unknown. Only limited numbers of studies are published in the forms of case reports or short series of literature reviews. Hence, conclusive interpretations are not available (11–14). We performed a cohort study to explore the probable interaction between AOSD and pregnancy.

## Materials and Methods

### Patients

This cohort study was conducted in the Department of Rheumatology and Immunology, Ruijin Hospital, Shanghai Jiao Tong University School of Medicine. Inclusion criteria were: (i) AOSD diagnosis fulfilling the Yamaguchi criteria; (ii) exclusion diagnosis including infections, malignancies, and other systemic immune diseases; (iii) inpatients and outpatients of Rheumatology and Immunology, Ruijin Hospital from September 2015 to March 2019; and (iv) female AOSD patients reported at least one pregnancy. This study was approved by the Ethics Committee of Ruijin Hospital, Shanghai Jiao Tong University (ID: 2016-61). According to the Declaration of Helsinki, informed consent was obtained from each patient.

### Data Collection

Data on gestation history included age at pregnancy, gravidity, pregnancy outcomes, comorbidities, and obstetric complications. Clinical characteristics of AOSD and medication usage during pregnancy were collected from medical records and questionnaires interviewed by a research team member. Pregnancies were categorized as pre-AOSD (defined as delivery at least 12 months before AOSD onset), post-AOSD (pregnancy after AOSD diagnosis), gestational AOSD (defined as AOSD onset during pregnancy), and postpartum AOSD (defined as AOSD onset within 1 year after delivery). Exposure variables included temporal relationship with AOSD, maternal age, gravidity (defined as the total number of previous pregnancies), disease activity (determined by the presence of fever and/or any suggestive cutaneous and/or inflammatory arthralgia/arthritis and/or sore throat), and conception-disease interval (defined as the interval time between disease onset and conception). The pregnancy outcomes included normal delivery, induced abortion, induced labor, preterm birth (PTB, defined as the pregnancy ended between 28 and 37 weeks), full-term cesarean section (CS, defined as the use of surgery for delivery after 37 weeks), and spontaneous abortion (STA, defined as spontaneous embryonic/fetal loss prior to 20 weeks). The disease pattern of AOSD patients was divided into three distinct types: monocyclic, polycyclic, and chronic courses over a 12-month follow-up period (1).

### Statistical Analysis

We performed the Mann-Whitney *U*-test for continuous variables and Fisher's exact test for categorical variables. A generalized random mixed-effect model was constructed with individuals as the random effect; temporal relation (categorical), maternal age (continuous), and gravidity (continuous) as the fixed effects. To remove the effects of confounding factors, we performed the propensity score matching method to compare the pregnancy outcome between pre-AOSD and post-AOSD groups. Besides, we also performed a dependent sample sign test to evaluate whether disease relapse increased during pregnancy.

A two-sided *p* < 0.05 was considered statistically significant. Quantitative variables were expressed as median (IQR) and categorical variables were presented as frequency (percentage). Statistical analysis was performed with the IBM SPSS Statistics for Windows, version 22.0 (IBM Corp., Armonk, N.Y., USA) and R statistical software version 3.5.2.

## Results

A total of 86 patients were enrolled in this study. The clinical manifestations of 86 patients at the time of AOSD diagnosis are presented in Supplementary Table 1, the median age at AOSD onset was 37 years, and the median of disease duration was 18 months. The most common clinical manifestations were fever (100%), skin rash (91.9%), and arthralgia (88.4%) at disease onset. Among the 191 pregnancies, 164 (85.9%) occurred before AOSD, 16 (8.4%) after AOSD, three (1.6%) coincided with the onset of AOSD, and eight (4.2%) were postpartum AOSD (Table 1). All the conceptions were natural. The median age at pregnancy onset was 24 years, which is similar to the nationwide sample survey (15). No patients had the history of smoking or drinking. The comorbidities of pregnancy included hypertension, diabetes mellitus, and thyroid dysfunction. There was no previous record of intrauterine growth restriction or premature rupture of membranes during pregnancy in our study. Conception-disease interval was 234.0 months in pre-AOSD, 30.0 months in post-AOSD, 2.0 months in gestational AOSD, and 13.0 months in postpartum AOSD. Therapeutic strategies before and during pregnancy were also shown in Table 1. Hydroxychloroquine (31.3%) and glucocorticoids (18.8%) were most frequently selected in post-AOSD group. The detailed treatment information was shown in Supplementary Table 2.

**Table 1:** Characteristics of 191 pregnancies grouped by temporal relationship between disease onset and pregnancy.

**Variable**	**Total**	**Pre-AOSD**	**Post- AOSD**	**Gestational AOSD**	**Postpartum AOSD**
Patients, *n* (%)	86 (100.0)	79 (91.9)	13 (15.1)	3 (3.5)	8 (9.3)
Pregnancy episodes, *n* (%)	191 (100.0)	164 (85.9)	16 (8.4)	3 (1.6)	8 (4.2)
Gravidity, median (IQR)	2 (1–2)	2 (1–2)	2 (1–4)	2 [1–3]^*^	2 (1–2)
Comorbidities during pregnancy, *n* (%)					
Hypertension	1 (0.5)	0 (0.0)	1 (6.3)	0 (0.0)	0 (0.0)
Diabetes mellitus	1 (0.5)	1 (0.6)	0 (0.0)	0 (0.0)	0 (0.0)
Thyroid dysfunction	1 (0.5)	0 (0.0)	1 (6.3)	0 (0.0)	0 (0.0)
Conception-disease interval^#^, months, median (IQR)	168.0 (48.0–336.0)	234 (84.0–348.0)	30.0 (22.8–45.6)	2.0 [1.8–3.0]^*^	13.0 (11.2–15.5)
Treatment before pregnancy^Δ^					
No medication	185 (96.9)	164 (100.0)	10 (62.5)	3 (100.0)	8 (100.0)
Glucocorticoids	3 (1.6)	–	3 (18.8)	–	–
Methotrexate	1 (0.5)	–	1 (6.3)	–	–
Cyclosporine	2 (1.0)	–	2 (12.5)	–	–
Hydroxychloroquine	5 (2.6)	–	5 (31.3)	–	–
Treatment during pregnancy					
No medication	183 (95.8)	164 (100.0)	10 (62.5)	1 (33.3)	8 (100.0)
Glucocorticoids	5 (2.6)	–	3 (18.8)	2 (66.7)	–
Methotrexate	1 (0.5)	–	1 (6.3)	0 (0.0)	–
Cyclosporine	2 (1.0)	–	2 (12.5)	0 (0.0)	–
Hydroxychloroquine	5 (2.6)	–	5 (31.3)	0 (0.0)	–

*Qualitative variables were expressed as n (%); continuous variables were expressed as median (IQR); AOSD, Adult-onset Still's disease*.

# *Conception-disease interval was defined as the interval between disease onset and conception*.

* *Presented as [min, max]*.

Δ *The medication within three months before pregnancy was recorded*.

Regarding the two major groups: pre-AOSD and post-AOSD group, the post-AOSD group had significantly older maternal age (29 years [26–32 years] vs. 25 years [23–28 years], respectively; *p* = 0.005), a lower proportion of normal delivery (25.0 vs. 52.4%, *p* = 0.036) and a higher proportion of STA (18.8 vs. 0.6%, *p* = 0.002) compared with the pre-AOSD group. Obstetric complications and neonatal situation showed no significant difference between the two major groups (Table 2). In order to reduce the interference of confounding factors and potential biases, we performed the propensity score matching method stratified by maternal age and gravidity (Supplementary Figure 1). Following propensity score matching, the post-AOSD group still had a significantly lower proportion of normal delivery (20.0 vs. 50.0%, *p* = 0.045) and a higher proportion of STA (20.0 vs. 0.0%, *p* = 0.017) compared with the pre-AOSD group (Supplementary Table 3).

**Table 2:** Maternal characteristics of pregnancies in women that occurred before and after AOSD diagnosis.

	**Pre-AOSD**	**Post-AOSD**	***p-*value**
Pregnancy episodes, *n*	164	16	
Age at pregnancy onset, median (IQR), years	25 (23–28)	29 (26–32)	0.005
Duration of pregnancy, median (IQR), weeks	40 (39–40)	40 (39–40)	0.944
Gravidity, median (IQR)	2 (1–2)	2 (1–4)	0.057
Pregnancy outcomes, *n* (%)			
Normal delivery	86 (52.4)	4 (25.0)	0.036
Induced abortion	50 (30.5)	5 (31.2)	1.000
STA	1 (0.6)	3 (18.8)	0.002
Full-term CS	23 (14.0)	4 (25.0)	0.268
Induced labor, *n* (%)	3 (1.8)	0 (0.0)	0.497
PTB	1 (0.6)	0 (0.0)	1.000
Obstetric complications, *n* (%)			
Oligohydramnios	1 (0.6)	0 (0.0)	1.000
Antepartum hemorrhage	5 (3.0)	0 (0.0)	0.246
Length of hospital stay, median (IQR), days	4 (4–5)	4 (3–7)	0.869
Neonatal situation			
Birth weight, median (IQR), kg	3.4 (3.0–3.8)	3.6 (2.9–3.9)	0.655
Neonatal death, *n* (%)	1 (0.6)	0 (0.0)	1.000
AOSD features			1.000^*^
Flare		1 (6.3)	
Remission		15 (93.7)	

*Pre-AOSD, delivery at least 12 months before AOSD diagnosis; Post-AOSD, pregnancy after AOSD diagnosis; PTB, preterm birth; STA, spontaneous abortion; CS, cesarean section; AOSD, Adult-onset Still's disease; IQR, interquartile range*.

*Mann-Whitney U-test was used for continuous variables and Fisher's exact test was used for categorical variables*.

* *A dependent sample sign test*.

In order to demonstrate whether AOSD could have any impact on pregnancy outcome independent of known risk factors including maternal age and gravidity, we constructed a generalized linear mixed effect model, with the binomial variable (pre- or post-AOSD) as fixed effect, and individual female patient as random effect. Pregnancy after AOSD was significantly associated with STA (adjusted OR = 4.577, 95% CI: 4.166–845.119; *p* = 0.003) (Table 3).

**Table 3:** The ORs of pregnancy outcomes in post- AOSD pregnancy.

**Pregnancy outcomes**	**Adjusted OR^**a**^**	**95% CI**	***p-*value**
Normal delivery	0.239	0.043–1.317	0.100
Induced abortion	0.670	0.198–2.267	0.519
STA	4.577	4.166–845.119	0.003
Full term CS	2.894	0.485–17.278	0.244
Induced labor	Model undetectable		
PTB	Model undetectable		

a *Adjusted for maternal age and gravidity; OR, odds ratio; PTB, preterm birth; STA, spontaneous abortion; CS, cesarean section; AOSD, Adult-onset Still's disease*.

*A generalized random mixed-effect model was constructed with individuals as the random effect; temporal relation (categorical), maternal age (continuous), and gravidity (continuous) as the fixed effects*.

In the post-AOSD group, the disease activity of 13 patients (16 pregnancies) at the time of conception was inactive. Eight patients didn't take any medication during gestation. The outcomes were one induced abortion, two STA, three full-term CS, and four normal delivery. Five patients (six pregnancies) were taking medications during gestation: low-dose glucocorticoids (3/6), hydroxychloroquine (5/6), cyclosporine (2/6), and methotrexate (1/6). Except one pregnancy ended up with induced abortion because of methotrexate treatment, the outcomes of the other five pregnancies were one STA, three induced abortion, and one full-term CS. Disease flare upon conception was observed in one out of 16 post-AOSD pregnancies. A dependent sample sign test deduced that disease activity was not exacerbated by pregnancy (*p* = 1.000) (Table 2) for the post-AOSD group.

Among the 86 patients, 3.5% (3/86) patients had disease onset during gestational period and 9.3% (8/86) during postpartum period. The detailed characteristics of pregnancies in gestational and postpartum groups were shown in Supplementary Table 2. In the gestational AOSD group (*n* = 3), two onsets of AOSD occurred in the first trimester of pregnancy, both achieved disease remission after induced abortion. Another onset of AOSD occurred in the 12th week of gestation, and the initial therapy of prednisone 40 mg/day failed to relieve the symptoms. Due to oligohydramnios, she underwent cesarean section and the symptoms were relieved. However, the disease flared 3 months after delivery and complicated with macrophage activation syndrome (MAS). She was treated with dexamethasone and etoposide (VP16), followed by cyclosporine. The postpartum group included eight patients (8 pregnancies), the medications used including median-dose glucocorticoids (3/8), high-dose glucocorticoids (5/8), cyclosporine (4/8), methotrexate (8/8), etanercept (1/8), and hydroxychloroquine (7/8). Over a 12-month follow-up period, the disease courses of gestational and postpartum groups (*n* = 11) evolved into monocyclic course (27.3%), polycyclic course (45.4%), and chronic course (27.3%).

## Discussion

Akin to lots of autoimmune diseases with a strong female preponderance, relationships between pregnancy and consequent development of AOSD have been the source of studies, even though they usually showed conflicting results (16). Here, we conducted a cohort study, which might provide the largest amount of information on pregnancy and AOSD. Our data showed that AOSD increased more than 4-fold the odds of STA during pregnancy, while pregnancy might not trigger the relapse of AOSD. Besides, new-onset AOSD during gestation and postpartum tended to evolve into the polycyclic course.

The reoccurrence of AOSD during pregnancy was first reported in 1980 (17). Le Loët et al. reported five pregnancies in four AOSD patients including two diagnosed AOSD. They found that pregnancy had no adverse effect on AOSD, and AOSD had no influence on pregnancy outcomes (11). On the contrary, adverse influence of pregnancy on AOSD has also been reported, especially during the first, second trimester, and postpartum period. In 2004, Mok et al. reported the maternal and fetal outcomes of five pregnancies in three AOSD patients (12). Even though all of them were in medicine-free remission at conception, an exacerbation occurred in the fourth and fifth months of gestation and during the postpartum period. In 2012, Yamamoto et al. reviewed 23 pregnancies in 19 AOSD women (18). There were nine patients who developed the onset of AOSD during pregnancy, and most occurred in the second trimester until puerperium. In our study, 3.5% (3/86) AOSD patients had their first AOSD-related manifestations during the gestational period and 9.3% (8/86) during the postpartum period. The high percent of AOSD diagnosis during the gestational and postpartum periods suggest that sex hormones may increase the risk of new-onset AOSD. In 1971, Bywaters noticed the female predominance in AOSD indicating that sex hormones might influence the disease susceptibility (19). The underlying biological mechanism is not clear. Previous study reported that estrogens could activate macrophages to produce tumor necrosis factor (TNF-α), IL-6, and IL-1 (20). It could also boost the expression of IL-1 mRNA through monocytes as well as increase several aspects of endothelial-cell biological functions, such as adhesion to matrix proteins, migration and cell differentiation, and promoting inflammation (21). Besides, a recent research speculated that increased IL-18 during pregnancy may participate in the pathogenesis of the onset of AOSD (18, 22), which requires further studies to confirm.

However, only one diagnosed AOSD patient had disease relapse during pregnancy, which indicated that AOSD will not be commonly exacerbated by pregnancy if it is well-controlled. The impacts of pregnancy on the disease onset are diverse with regard to different diseases. The flare rate of SLE increased during pregnancy and postpartum, ranging from 25 to 60% of pregnancies (23). Nevertheless, studies showed that 54–95% of patients with RA improved during pregnancy, with nearly 40% patients achieving a state of remission (24, 25). After delivery, there is an increased risk of a flare in disease activity of RA, postpartum exacerbations varied from 62 to 90% (26). These different flare rates between different rheumatic diseases are probably due to (1) sex hormones such as estrogen might play different role in different diseases (27). And, (2) maternal shift from a Th1 to Th2 immune response during pregnancy. What is more, Léo Plaçais et al. reviewed 19 AOSD cases during pregnancy. They found that none of the cases presented as a chronic articular form and no obvious difference was found between monocyclic and polycyclic patterns. Our results showed that new-onset AOSD during pregnancy and postpartum had a higher percent to evolve into the polycyclic course (45.4%), which is different from the previous study.

It is still unknown whether AOSD can lead to poor pregnancy outcomes. Previously, Leo reported a case-based review gathering data about 19 cases of AOSD revealed during pregnancy (28). The obstetrical complications occurred in nearly 50% of AOSD patients including prematurity (10/20), pre-term premature rupture of membranes (3/20), intrauterine growth restriction (3/20), oligohydramnios (2/20), or neonatal death (1/20). In our study, 18.8% (3/16) of diagnosed AOSD had STA, and the generalized linear mixed model and propensity score matching method demonstrated that AOSD contributed to an increased risk of STA. In addition, one patient in gestational group underwent cesarean section due to oligohydramnios, however, the patient was not exposed to any NSAID, which was reported to be associated with low amniotic fluid levels (29). Besides, treatment during pregnancy may increase the incidence of adverse outcome. Some studies indicated that gestational exposure to corticosteroids led to a slightly increased risk of premature birth (30). In our study, one patient underwent induced abortion for fear of abnormal fetal development resulting from methotrexate exposure during pregnancy. As a result, drug factors should be taken into account cautiously.

The treatment of AOSD during pregnancy is challenging. Glucocorticoids are a mainstay of treatment for patients with AOSD (31, 32), despite the potential increased risk of gestational diabetes, arterial hypertension, intrauterine growth restriction, or pre-term premature rupture of membranes (33–36). Intravenous immunoglobulin (IVIG) has been reported for the management of AOSD during pregnancy (37), especially for the life-threatening complications. Recently, Smith reported Anakinra was successfully used in five patients during pregnancy with no serious complications or adverse pregnancy outcomes (38), which provided alternative therapies for pregnancy-related AOSD.

Currently, our study provided the largest sample of AOSD patients to clarify the relationship between pregnancy and AOSD. The enrolled patients had a similar gestational age to the reported distribution of the nationwide survey, which makes our sample more representative. What is more, through a mixed effect logistic regression model, we found post-AOSD is robust associated with STA. We also performed the propensity score matching method to reduce the effects of confounding in our observational study. However, there are still some limitations. The fact that results only from single tertiary center give the generalizability many limitations, so multi-centered studies should be conducted in order to further confirm the interaction between pregnancy and AOSD. Moreover, the follow-up sample about gestational and postpartum groups is too small to reach a definitive conclusion of disease evolution; as a result, prospective studies with a larger sample size are needed in the future.

## Conclusions

In summary, we found that pregnancy in patients diagnosed with AOSD was associated with an increased risk of complicated pregnancies, which should be anticipated by both rheumatologists and obstetricians. However, the disease activity of AOSD was not exacerbated by pregnancy if it was well-controlled, which emphasizes the importance of disease evaluation before pregnancy.

## Data Availability Statement

The original contributions presented in the study are included in the article/Supplementary Materials, further inquiries can be directed to the corresponding authors.

## Ethics Statement

The studies involving human participants were reviewed and approved by Ethics Committee of Ruijin Hospital, Shanghai Jiao Tong University (ID: 2016-61). According to the Declaration of Helsinki, informed consent was obtained from each patient.

## Author Contributions

ZW and HC performed statistical analysis and drafted the manuscript. QD, TF, LW, JT, QH, JJ, TL, XW, and ZZ collected the data. TZ, HL, XC, JY, HS, and YSun extracted the data. CY and YSu conceived the study and contributed to discussion. All authors reviewed and approved the final manuscript.

## Conflict of Interest

The authors declare that the research was conducted in the absence of any commercial or financial relationships that could be construed as a potential conflict of interest. The reviewer SY declared a shared affiliation, though no other collaboration, with several of the authors ZW, HC, TF, QD, JT, HL, XC, JY, HS, YSun, CY, ZZ, XW, LW, TL, JJ, QH, and YSu to the handling editor.
