# Supplementary material for: Pregnancy Outcomes in Patients With Adult-Onset Still's Disease: A Cohort Study From China
Source: Front Med (Lausanne). 2020 Dec 8;7:566738. doi: 10.3389/fmed.2020.566738 (PMC7753176; doi:10.3389/fmed.2020.566738)
Supplement: Supplementary file 1 [file Table_1.docx]

**Supplementary Table 1.** Baseline characteristics of the AOSD patients at the time of diagnosis.

| **Patients characteristics** | **All (n = 86)** |
| --- | --- |
| Age at pregnancy onset, median (IQR), years | 24 (22-26) |
| Age at AOSD onset, median (IQR), years | 37 (29-52) |
| Disease duration at enrollment, median (IQR), years | 18 (7-36) |
| Clinical features at AOSD diagnosis, n (%) |  |
| Fever | 86 (100.0) |
| Sore throat | 60 (69.8) |
| Skin rash | 79 (91.9) |
| Lymphadenopathy | 62 (72.1) |
| Splenomegaly | 34 (39.5) |
| Hepatomegaly | 6 (7.0) |
| Pericarditis | 17 (19.8) |
| Pleuritis | 17 (19.8) |
| Myalgia | 26 (30.2) |
| Pneumonia | 28 (32.6) |
| Arthralgia | 76 (88.4) |
| Arthritis | 49 (57.0) |
| Systemic score | 5.0 (4.0-6.0) |
| Laboratory markers at AOSD diagnosis, median (IQR) |  |
| Hemoglobin, g/L | 109.0 (92.8-120.0) |
| Leukocytes, 10^9/L | 16.3 (5.4-22.2) |
| Platelets, 10^9/L | 258.0 (188.0-334.0) |
| ESR, mm/h | 52.0 (22.5-77.0) |
| CRP, mg/L | 40.0 (11.7-99.0) |
| ALT, U/L | 28.0 (16.0-43.5) |
| AST, U/L | 41.5 (26.3 - 67.0) |
| Ferritin, ng/mL | 3241.2 (324.1-6754.3) |
| Inflammatory markers at AOSD diagnosis, median (IQR) |  |
| IL-1β (pg/ml) | 0.1 (0.1-0.3) |
| IL-6 (pg/ml) | 1.6 (0.6-7.6 ) |
| TNF-α (pg/ml) | 4.8 (3.6-8.0) |
| IL-18 (ng/ml) | 101.3 (24.7-211.2) |

AOSD, adult-onset Still’s disease; IQR, interquartile range; ESR, erythrocyte sedimentation rate; CRP, C-reactive protein; AST, aspartate transaminase; ALT, alanine transaminase; IL, interleukin.
