# Supplementary material for: Pregnancy Outcomes in Patients With Adult-Onset Still's Disease: A Cohort Study From China
Source: Front Med (Lausanne). 2020 Dec 8;7:566738. doi: 10.3389/fmed.2020.566738 (PMC7753176; doi:10.3389/fmed.2020.566738)
Supplement: Supplementary file 2 [file Table_2.docx]

**Supplementary Table 2.** Disease activity and pregnancy outcomes of patients in post-AOSD, gestational AOSD, and postpartum AOSD group.

| **Patient** | **Age** | **AOSD features** | | **Treatment** | | | **Outcomes** | |
| --- | --- | --- | --- | --- | --- | --- | --- | --- |
|  |  | **Disease onset** | **Clinical manifestations** | **Before**  **pregnancy** | **During pregnancy** | **After**  **pregnancy*** | **Pregnancy outcomes** | **Disease course** |
| 1 | B.20 | 8^th^ week of gestation | F, R, J, ST, LN | None | None | Pred 30mg;  MTX; HCQ | Induced abortion | Polycyclic |
| 2 | C.30 | 7^th^ week of gestation | F, ST, R, M | None | Pred 60mg/d | Pred 60mg;  MTX; HCQ | Induced abortion | Monocyclic |
|  | D.31 | Post-AOSD | None | None | None | None | STA |  |
|  | E.32 | Post-AOSD | None | None | None | None | Full term CS |  |
| 3 | A.22 | 12^th^ week of gestation | F, R, J, ST, M, LN,  PC, PLE, HS, S | None | Pred 40mg | Dx 30mg;  CsA;VP16 | PTB | Polycyclic |
| 4 | B.26 | 4^th^ month postpartum | F, R, J, LN, SMG,  ST, M | None | None | Pred 40mg;  MTX; HCQ | Normal delivery | Polycyclic |
|  | C.28 | Post-AOSD | None | HCQ | HCQ | HCQ | Induced abortion |  |
|  | D.29 | Post-AOSD | None | HCQ | HCQ | HCQ | Induced abortion |  |
| 5 | A.19 | 6^th^ month postpartum | F, R, J, SMG, ALFT,  LN, ST, AP | None | None | Pred 160mg;  MTX; CsA; HCQ | Full term CS | Chronic |
| 6 | A.26 | 3^rd^ month postpartum | F, R, J, SMG, LN, ST,  M | None | None | Pred 160mg;  MTX; CsA; HCQ | STA | Chronic |
|  | B.30 | Post-AOSD | None | HCQ | HCQ | HCQ | Induced abortion |  |
| 7 | B.24 | 1^st^ month postpartum | F, J, HS | None | None | Pred 50mg;  MTX; HCQ; Etanercept | Induced abortion | Chronic |
| 8 | B.29 | 8^th^ month postpartum | F, R, J, ALFT, ST, M | None | None | Pred 120mg;  MTX; CsA | Induced abortion | Polycyclic |
| 9 | D.26 | 3^rd^ month postpartum | F, R, J, ALFT, SMG, LN,  ST, M, PC, PLE, P | None | None | Pred 120mg;  MTX; CsA; HCQ | Normal delivery | Polycyclic |
|  | E.29 | Post-AOSD | None | Pred 4mg;  CsA; HCQ | Pred 4mg;  CsA; HCQ | Pred 4mg;  CsA; HCQ | STA |  |
| 10 | B.32 | 2^nd^ month postpartum | F, R, J, ST, LN | None | None | Pred 40mg;  MTX; HCQ | Normal delivery | Monocyclic |
| 11 | B.20 | 1^st^ month postpartum | F, R, J, ALFT, P | None | None | Pred 120mg;  MTX; HCQ | Normal delivery | Monocyclic |
| 12 | A.23 | Post-AOSD | Remission | None | None | None | Full term CS | Polycyclic |
| 13 | B.38 | Post-AOSD | Remission | None | None | None | Normal delivery | Monocyclic |
| 14 | A.27 | Post-AOSD | Remission | Pred 15mg  CsA;HCQ | Pred 15mg  CsA;HCQ | Pred 15mg  CsA;HCQ | Full term CS | Monocyclic |
| 15 | A.25 | Post-AOSD | Remission | None | None | None | Normal delivery | Monocyclic |
|  | B.34 | Post-AOSD | Remission | None | None | None | Normal delivery |  |
| 16 | B.29 | Post-AOSD | Remission | None | None | None | Full term CS | Monocyclic |
| 17 | B.27 | Post-AOSD | Remission | None | None | None | Induced abortion | Monocyclic |
| 18 | C.22 | Post-AOSD | Remission | None | None | None | STA |  |
| 19 | B.37 | Post-AOSD | Remission | Pred 5mg/d,  MTX | Pred 5mg/d,  MTX | Pred 5mg/d,  MTX | Induced abortion | Monocyclic |
| 20 | A.22 | Post-AOSD | Remission | None | None | None | Normal delivery | Monocyclic |

A: first pregnancy; B: second pregnancy; C: third pregnancy; D: forth pregnancy; E: fifth pregnancy; Pred: prednisolone; Dx: dexamethasone; MTX: methotrexate; HCQ: Hydroxychloroquine; CsA: Cyclosporine;VP-16; Etoposide; F: fever; HS: hepatosplenomegaly; SMG: splenomegaly; LN: lymphadenopathies; R: rash; ST: sore throat; S: serositis; P: pneumonia; J: arthralgia; M: myalgia; ALFT: abnormal liver function tests; PC: pericarditis; PLE: pleuritis; AP: abdominal pain; PTB: preterm birth; STA: spontaneous abortion; CS: cesarean section; AOSD: Adult-onset Still’s disease.

* The maximum dose of glucocorticoids used.
