# Supplementary material for: Pregnancy Outcomes in Patients With Adult-Onset Still's Disease: A Cohort Study From China
Source: Front Med (Lausanne). 2020 Dec 8;7:566738. doi: 10.3389/fmed.2020.566738 (PMC7753176; doi:10.3389/fmed.2020.566738)
Supplement: Supplementary file 3 [file Table_3.docx]

**Supplementary Table 3.** Comparison of pregnancy outcomes between pre-AOSD group and post-AOSD group following propensity score matching.

| **Pregnancy outcomes** | **Pre-AOSD** | **Post-AOSD** | \| ***p* Value** \| \| --- \| |
| --- | --- | --- | --- | --- |
| Pregnancy episodes, n | 40 | 15 |  |
| Normal delivery, n (%) | 20(50.0) | 3(20.0) | 0.045 |
| Induced abortion, n (%) | 15(37.5) | 5(33.3) | 0.775 |
| STA, n (%) | 0(0.0) | 3(20.0) | 0.017 |
| Full term CS, n (%) | 4(10.0) | 4(26.7) | 0.193 |
| Induced labour, n (%) | 1(2.5) | 0(0.0) | 1.000 |
| PTB, n (%) | - | - | - |

Pre-AOSD: delivery at least 12 months before AOSD onset; Post-AOSD: pregnancy after AOSD diagnosis; PTB: preterm birth; STA: spontaneous abortion; CS: cesarean section; AOSD: Adult-onset Still’s disease.
