# Supplementary material for: Pregnancy Outcomes in Patients With Adult-Onset Still's Disease: A Cohort Study From China
Source: Front Med (Lausanne). 2020 Dec 8;7:566738. doi: 10.3389/fmed.2020.566738 (PMC7753176; doi:10.3389/fmed.2020.566738)
Supplement: Supplementary file 4 [file Data_Sheet_1.docx]

**Supplementary Figure 1.** Propensity scores matching method stratified by maternal age and gravidity.


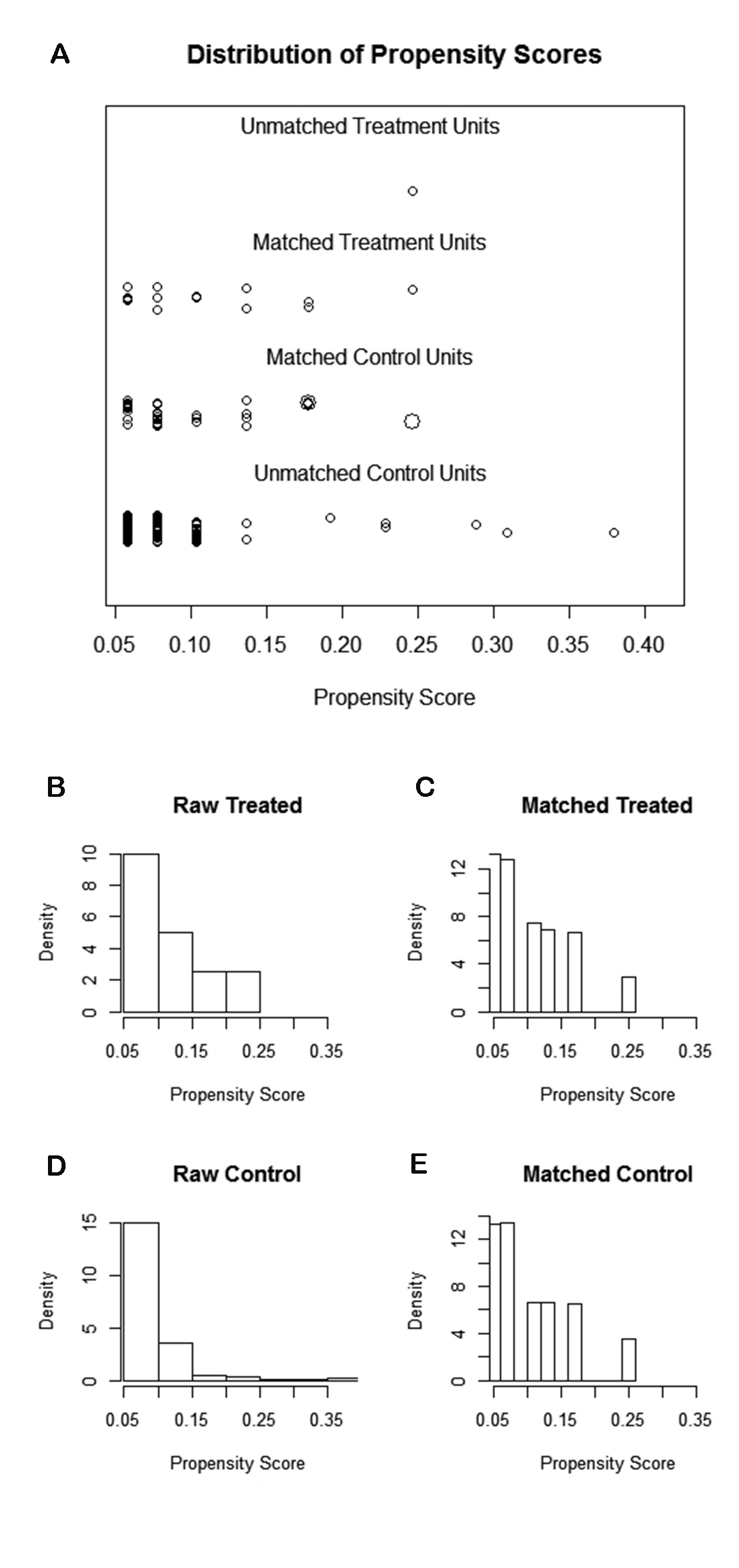


After 3-to-1 propensity score caliper matching out of 191 pregnancies, 55 pregnancies were selected for comparison: 40 pregnancies in pre-AOSD group and 15 pregnancies in post-AOSD group. (**A)** Distribution of propensity score matching jitter plot. (**B**-**E)** Histogram of propensity scores.
